# Selected Cytokines Serve as Potential Biomarkers for Predicting Liver Inflammation and Fibrosis in Chronic Hepatitis B Patients With Normal to Mildly Elevated Aminotransferases

**DOI:** 10.1097/MD.0000000000002003

**Published:** 2015-11-13

**Authors:** Yong-Qiong Deng, Hong Zhao, An-Lin Ma, Ji-Yuan Zhou, Shi-Bin Xie, Xu-Qing Zhang, Da-Zhi Zhang, Qing Xie, Guo Zhang, Jia Shang, Jun Cheng, Wei-Feng Zhao, Zhi-Qiang Zou, Ming-Xiang Zhang, Gui-Qiang Wang, China HepB Related Fibrosis Assessment Research Group

**Affiliations:** From the Department of Infectious Disease, Center for Liver Disease, Peking University First Hospital (Y-QD, HZ, J-YZ, G-QW); Department of Infectious Disease, China–Japan Friendship Hospital, Beijing (A-LM); Department of Infectious Disease, The Third Affiliated Hospital Sun Yat-Sen University, Guangzhou, Guangdong (S-BX); Department of Infectious Diseases, South West Hospital affiliated to Third Military Medical University (X-QZ); Department of Infectious Diseases, Second Affiliated Hospital of Chongqing Medical University, Chongqing (D-ZZ); Department of Infectious Diseases, Rui Jin Hospital Shanghai Jiao Tong University School of Medicine, Shanghai (QX); Department of Infectious Diseases, The People's Hospital of Guang Xi Zhuang Autonomous Region, Nanning, Guangxi (GZ); Department of Infectious Diseases, The People's Hospital Of He Nan province, Zhengzhou, Henan (J-S); Department of Infectious Diseases, Di Tan Hospital affiliated to Capital Medical University, Beijing (JC); Department of Infectious Diseases, Xinxiang Medical University Third Hospital, Xinxiang, Henan (W-FZ); Department of Infectious Diseases, Yantai City Hospital for Infectious Disease, Yantai, Shandong (Z-QZ); Department of Infectious Diseases, Shenyang Sixth People's Hospital, Shenyang (M-XZ); and Collaborative Innovation Center for Diagnosis and Treatment of Infectious Diseases, Zhejiang University, Hangzhou, Zhejiang; Liaoning, China (G-QW).

## Abstract

Previous studies of small cohorts have implicated several circulating cytokines with progression of chronic hepatitis B (CHB). However, to date there have been no reliable biomarkers for assessing histological liver damage in CHB patients with normal or mildly elevated alanine aminotransferase (ALT).

The aim of the present study was to investigate the association between circulating cytokines and histological liver damage in a large cohort. Also, this study was designed to assess the utility of circulating cytokines in diagnosing liver inflammation and fibrosis in CHB patients with ALT less than 2 times the upper limit of normal range (ULN).

A total of 227 CHB patients were prospectively enrolled. All patients underwent liver biopsy and staging by Ishak system. Patients with at least moderate inflammation showed significantly higher levels of CXCL-11, CXCL-10, and interleukin (IL)-2 receptor (R) than patients with less than moderate inflammation (*P* < 0.001). Patients with significant fibrosis had higher levels of IL-8 (*P* = 0.027), transforming growth factor alpha (TGF-α) (*P* = 0.011), IL-2R (*P* = 0.002), and CXCL-11 (*P* = 0.032) than the group without significant fibrosis. In addition, 31.8% and 29.1% of 151 patients with ALT < 2 × ULN had at least moderate inflammation and significant fibrosis, respectively. Multivariate analysis demonstrated that CXCL-11 was independently associated with at least moderate inflammation, and TGF-α and IL-2R independently correlated with significant fibrosis in patients with ALT < 2 × ULN. Based on certain cytokines and clinical parameters, an inflammation-index and fib-index were developed, which showed areas under the receiver operating characteristics curve (AUROC) of 0.75 (95% CI 0.66–0.84) for at least moderate inflammation and 0.82 (95% CI 0.75–0.90) for significant fibrosis, correspondingly. Compared to existing scores, fib-index was significantly superior to aspartate aminotransferase (AST) to platelet ratio index (APRI) and FIB-4 score for significant fibrosis.

In conclusion, CXCL-11 was independently associated with at least moderate inflammation, whereas IL-2R and TGF-α were independent indicators of significant fibrosis in both, total CHB patients and patients with normal or mildly elevated ALT. An IL-2R and TGF-α based score (fib-index) was superior to APRI and FIB-4 for the diagnosis of significant fibrosis in patients with normal or mildly elevated ALT.

## Abstract 2

Supplemental Digital Content is available in the text

## INTRODUCTION

Chronic HBV infection affects 350 to 400 million individuals globally.^1,2^ Each year, approximately 50,000 people die from HBV-related cirrhosis and hepatocellular carcinoma.^3^ Chronic HBV infection is a serious health problem in several countries, including China where 7.2% people have the condition.^4^

Alanine aminotransferase (ALT) has been widely used for assessing hepatitis activity. However, patients with normal or mildly elevated serum ALT are not necessarily free from liver damage or liver-related mortality.^5^ Under current guidelines, treatment decision for patients with normal or mildly elevated ALT (ALT < 2 × ULN, upper limit of normal range) depend on hepatic histology.^6,7^ Although liver biopsy remains the main method to determine the severity of liver injury, it is an invasive procedure associated with rare, but serious complications. The limitations of liver biopsy have led to the development of noninvasive assessments for histological liver damage. Nevertheless, there are no reliable biomarkers or noninvasive models for assessing liver inflammation and fibrosis in chronic hepatitis B (CHB) patients with normal or mildly elevated ALT.

Recently, emerging evidence has suggested that cytokines and regulatory molecules are fundamental mediators in the host adaptive immune response to HBV and viral clearance.^8^ Cytokines are a large family of molecules, including T helper type 1 cells (Th1) cytokines (interleukin-2 [IL-2], interferon [IFN]-γ, tumor necrosis factor-alpha [TNF-α], IL-12, IL-18), which enhance the cellular immune responses, Th2 type cytokines (IL-4, IL-6, IL-10, IL-13), which favor antibody responses, and Th17 cytokines (IL-17, IL-22, IL-23, IL-1, TNF-α) that contribute to the proinflammatory reaction.^8,9^ It has been proved in recent experimental studies that an imbalance in cytokine production plays a key role in the development of liver damage, necro-inflammation, and subsequent fibrosis. Specifically, IL-1 and TNF-α have been suggested to be highly proinflammatory cytokines, which contribute to hepatocyte apoptosis and immume cell activation. In addition, IL-20, IL-13, and IL-33 have been defined as profibrogenic cytokines that promote activation and proliferation of hepatic stellate cells.^10^ IL-22, which is produced primarily by Th17 and IL-22+ cells, has been reported to be largely increased in liver tissue of HBV-infected patients with liver cirrhosis.^11,12^ IL-17 signaling has been shown to promote production of IL-6, IL-1, and TNF-α by inflammatory cells and to directly induce production of collagen type I in hepatic stellate cells by activating the signal transducer and activator of transcription 3 (Stat3) signaling pathway.^13^

A few studies have also tried to reveal the possible correlation between circulating cytokines and histological liver damage in CHB patients. The current literature indicates that circulating Th17 cells increase histological activity index (HAI) via IL-17, IL-10, and INF-γ, and that IL-10, TNF-α, and transforming growth factor (TGF)-beta are correlated with fibrosis stages in CHB patients.^14–18^ In a cohort of African-American injection drug users with chronic hepatitis C, CXCR3 ligands (CXCL-11, CXCL-10, and CXCL-9) were shown to contribute to liver cirrhosis and to discriminate advance liver fibrosis from mild fibrosis.^9,19^ These studies, however, have included relatively few CHB patients with liver biopsy. Thus, larger CHB cohort studies are needed to further understand of the association between circulating cytokines and the pathological state of HBV-infected liver.

The aim of the current study was to investigate the association of circulating cytokines with liver inflammation and fibrosis in a large cohort. Also, this study was designed to assess the utility of circulating cytokines in predicting histological liver injury in CHB patients with normal or <2 × ULN ALT levels.

## MATERIALS AND METHODS

### Patients

A total of 235 patients were prospectively enrolled in this study. Inclusion criteria consisted of: positive hepatitis B surface antigen for at least 6 months; treatment naive; age between 18 and 65 years; negative serum levels for anti-HAV IgM, anti-HCV, anti-HEV IgM/IgG, anti-EBV IgM, and anti-CMV IgM; and off potential transaminase-lowering agents such as bicyclol for at least 2 weeks prior to blood sampling biochemistries. Patients were recruited from 24 hospitals in mainland China between October, 2013 and September, 2014. Exclusion criteria were: hepatitis C virus or human immunodeficiency virus coinfection; presence of other causes of chronic liver diseases such as alcoholic, autoimmune, genetic, drug-induced, and nonalcoholic fatty; and pregnancy. Patients with hepatocellular carcinoma or decompensated cirrhosis were also excluded. Clinical data and chemical parameters were documented, and serum samples were taken at the time of liver biopsy. Noninvasive assessments for fibrosis, aspartate aminotransferase to platelet ratio index (APRI), and FIB-4 score were calculated as follows: APRI = (aspartate aminotransferase [AST/ULN]/[platelet count × 10^9^/L]) × 100; FIB-4 = (age × AST)/(platelet count × 10^9^/L × ALT^1/2^).^20,21^ The reference value for ALT was 0–40 IU/L.^22^

All patients gave written informed consent for the use of data and samples for scientific purposes, and the study was approved by The Ethical Committees of Peking University First Hospital, China–Japan Friendship Hospital, The Third Affiliated Hospital Sun Yat-Sen University, South West Hospital affiliated to Third Military Medical University, Second Affiliated Hospital of Chongqing Medical University, Rui Jin Hospital Shanghai Jiao Tong University School of Medicine, The People's Hospital of Guang Xi Zhuang Autonomous Region, The People's Hospital of He Nan province, Di Tan Hospital affiliated to Capital Medical University, Xinxiang Medical University Third Hospital, Yantai City Hospital for Infectious Disease, Shenzhen Third People's Hospital, Public Health Clinical Center Affiliated to Fudan University, Third Hospital of Qin Huang Dao, Shenyang Sixth People's Hospital, Jiang Su Province Hospital, Dailian Sixth People's Hospital, Dalian, West China School of Medicine/West China Hospital Sichuan University, 302 Military Hospital of China, The First Hospital of Zhe Jiang Province, 305 Military Hospital of China, Beijing, Guangzhou Eighth People's Hospital, Eight-one Military Hospital of China, Nanjing, and The First Affiliated Hospital of Anhui Medical University. The full, detailed clinical trial protocol has been registered (NCT01962155 and ChiCTR-DDT-13003724).

###  HBV Serum Markers Measurement

Serum HBV DNA (dynamic range 2.0 × 10^1^–1.7 × 10^8^ IU/mL) was measured by COBAS AmpliPrep/COBAS TaqMan as previously described.^22^ Qualitative detection of hepatitis B core antigen (HBeAg) and HBeAb were also performed using appropriate Roche Elecsys assays according to the manufacture's instructions.

### Cytokines Detection

Fifteen cytokines (IL-1β, IL-2, IL-4, IL-6, IL-8, IL-10, IL-13, IL-17A, CCL-2, IL-12 p70, CCL-3, IFN-γ, TNF-α, TGF-α, and granulocyte monocyte colony stimulating factor) were measured by Human cytokines/Chemokine panel I (Cat. No. HCYTOMAG-60K, EMD Millipore, Billerica, MA). Six cytokines and chemokines (CXCL9, CXCL-10, CXCL11, IL-2R, IL-33, and IL-34) were measured by Luminex screening system (LXSAHM-6, R&D, Minneapolis, MN). The results were analyzed by a Luminex 200 system (EMD Millipore, Billerica, MA) according to the manufacturer's instructions. The coefficient of variation between the duplicate wells was controlled within 10%, and *R*^2^ of the standard curve was at least 0.999.

### Histological Staging

Ultrasonographic-guided liver biopsies were routinely processed in each institute according to a standardized protocol after receiving the patient's written informed consent. Specimens were fixed, paraffin-embedded, and stained with hematoxylin–eosin and Masson's trichrome. A minimum of 2.0 cm of liver tissue with at least 11 portal tracts were required for diagnosis. Pathological interpretations were conducted in the Department of Pathology at the You An Hospital affiliated to Capital Medical University. Each section was assessed by 2 pathologists, blindly and independently. When discrepancies occurred, the samples were reviewed by experienced pathologists who were also responsible for the reassessment of 10% random samples. All pathologists were blinded to the protocol. HAI and staging of liver fibrosis were assessed according to Ishak criteria.^23^ For analysis, HAI ≥ 5 was defined as moderate/significant inflammation, and F ≥ 3 as significant fibrosis.^24,25^

### Statistical Analysis

Statistical analyses were performed using SPSS ver. 16.0 (SPSS, Chicago, IL). Quantitative variables were expressed as the mean ± standard deviation (SD). Categorical variables were compared using the Chi-square test, and continuous variables were compared using the Mann–Whitney *U* test. A binary backward stepwise logistic regression analysis was conducted to determine the independent variables of liver inflammation and fibrosis. Receiver operating characteristic curves were created for the assessment of the predictive scores for staging inflammation and fibrosis. The predictive value of variables for severity of liver damage expressed as the area under the receiver operating characteristic curves curve (AUC), sensitivity, specificity, positive predictive value (PPV), and negative predictive value (NPV) were calculated. All *P*-values reported are 2-sided, and *P* < 0.05 was considered to be statistically significant. The classification accuracy of variables for diagnosis was validated by leave-one out cross-validation (LOOCV).

## RESULTS

### Clinical Characteristics

A total of 235 patients were enrolled in this study, and 8 patients were excluded due to inadequate liver tissue. The remaining 227 cases were analyzed, which included 151 with ALT < 2 × ULN and 76 with ALT ≥ 2 × ULN. Patients were also stratified by HBV DNA levels and HBeAg status. In patients with ALT ≥ 2 × ULN, 48 (63.2%) patients had at least moderate inflammation and 23 (30.3%) had significant fibrosis. Of 151 patients with ALT < 2 × ULN, 31.7% and 29.1% showed at least moderate inflammation and significant fibrosis, respectively. Clinical characteristics of the study population were shown in Table 1. Levels of 4 of 21 cytokines (IL-2, IL-4, IL-12 p70, and CXCL-9) were below the limits of detection in the sera of CHB patients. The remaining 17 cytokines were detected.

**TABLE 1:**
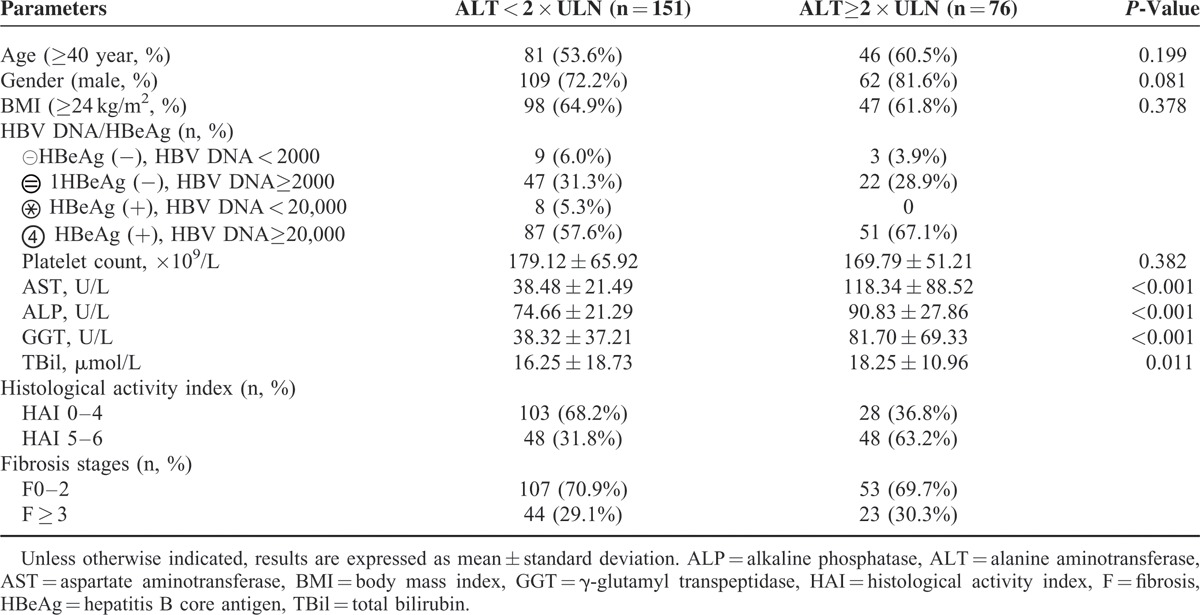
Clinical Characteristics of Patients With Chronic Hepatitis B Virus Infection

### Correlation Between Parameters, Cytokines, and Liver Inflammation

**FIGURE 1:**
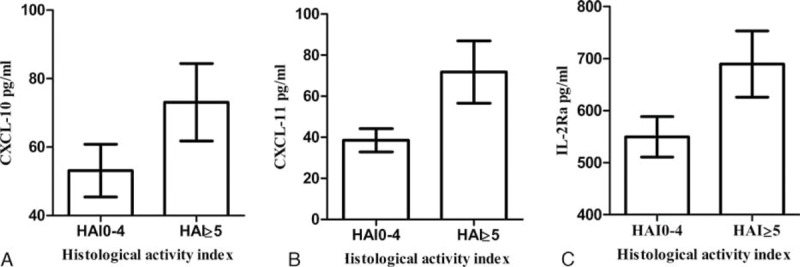
(A–C) Correlation between selected cytokines and histological scores of activity (n = 227). Median values with 95% confidence interval (of median) presented.

In the total CHB patient sample, there was no obvious association found between age, gender, body mass index (BMI), HBV DNA/HBeAg status, and liver inflammation (*P* > 0.05). Biochemical markers ALT (*P* < 0.001), AST (*P* < 0.001), alkaline phosphatase (ALP, *P* = 0.002), γ-glutamyl transpeptidase (GGT, *P* < 0.001), and total bilirubin (TBil, *P* = 0.043) were significantly elevated in patients diagnosed with at least moderate inflammation (Supplemental Table S1, http://links.lww.com/MD/A510). Cytokines and chemokine levels also correlated with HAI scores. Patients with moderate or severe inflammation had significantly higher levels of CXCL-11 (71.77 ± 75.06 pg/mL), CXCL-10 (73.08 ± 55.81 pg/mL), and IL-2R (689.47 ± 313.85 pg/mL) than the patients with no/mild inflammation (all *P* < 0.001, Figure 1). IFN-γ and IL-17A levels showed a declining trend with rising HAI scores (*P* = 0.066 and 0.076, respectively; Supplemental Table S1, http://links.lww.com/MD/A510) Multivariate analysis indicated that CXCL-11 (*P* = 0.009, OR = 1.012) and GGT (*P* < 0.001, OR = 1.017) were independently associated with moderate and severe inflammation (Table 2).

**TABLE 2:**
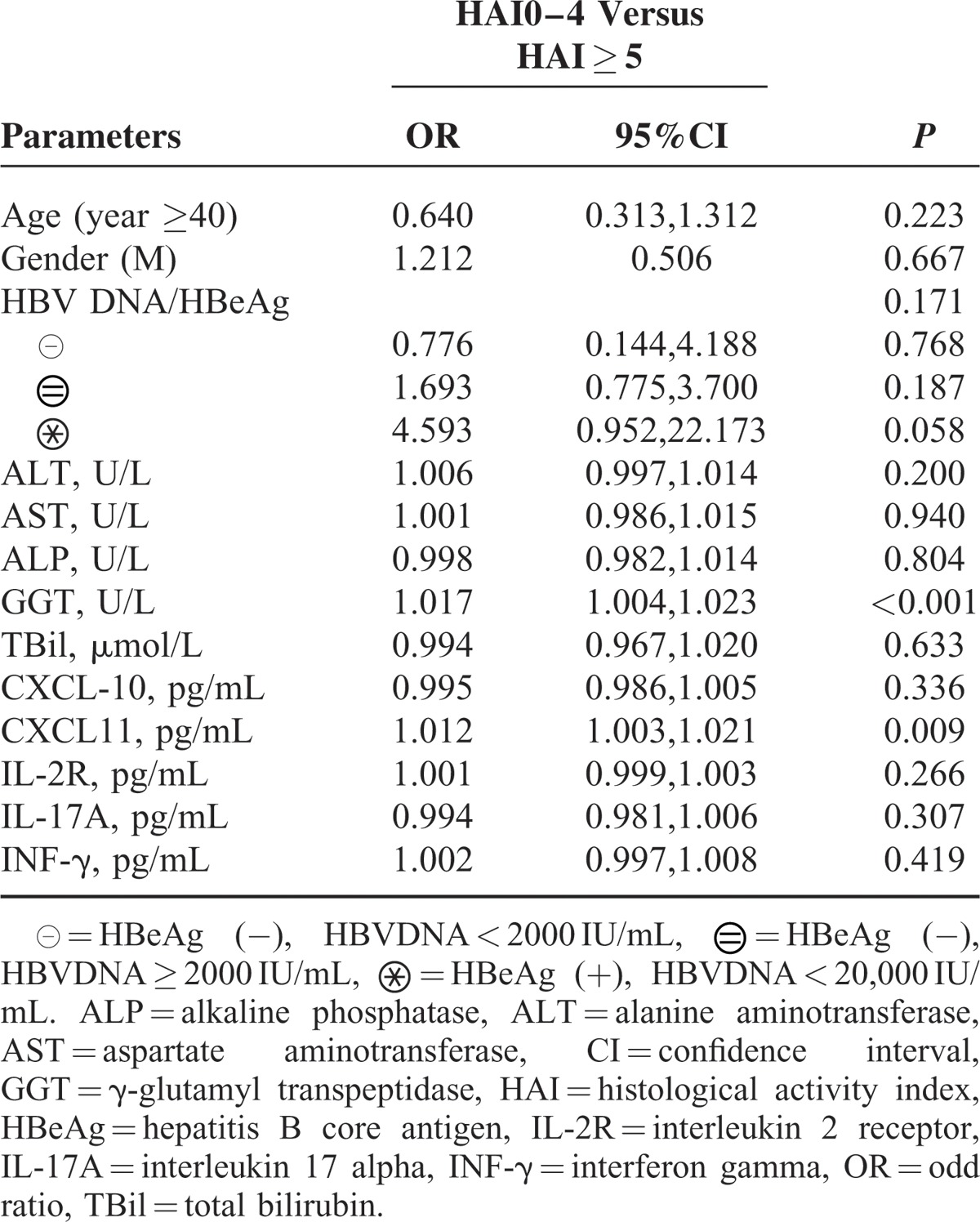
Multivariate Analysis of Clinical Parameters, Cytokines With Liver Inflammation in Total Patients (n = 227)

### Correlation Between Parameters, Cytokines, and Liver Fibrosis

**FIGURE 2:**
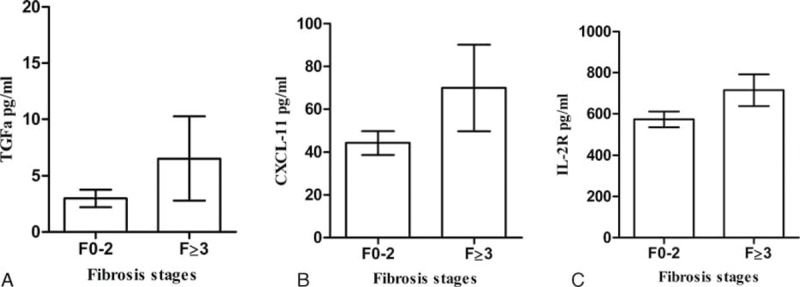
(A–C) Correlation between selected cytokines and histological fibrosis stages (n = 227). Median values with 95% confidence interval (of median) presented.

Platelet counts (PLT) were significantly decreased and GGT increased in patients with significant fibrosis. Age, gender, BMI, HBV DNA/HBeAg status, and ALT, AST, ALP, and TBil levels were not associated with fibrosis stages (*P* > 0.05). Patients with significant fibrosis had higher levels of IL-8 (*P* = 0.027), TGF-α (*P* = 0.011), CXCL-11 (*P* = 0.032), and IL-2R (*P* = 0.002) than patients with no significant fibrosis (Fig. 2). CXCL-10 showed an increasing trend in patients with significant fibrosis (*P* = 0.097, Supplemental Table S1, http://links.lww.com/MD/A510). Multivariate analysis showed that levels of TGF-α (*P* = 0.005, OR = 1.064), IL-2R (*P* = 0.008, OR = 1.002), CXCL-10 (*P* = 0.038, OR = 0.990), and PLT (*P* < 0.001, OR = 0.983) were independently associated with significant fibrosis (Table 3).

**TABLE 3:**
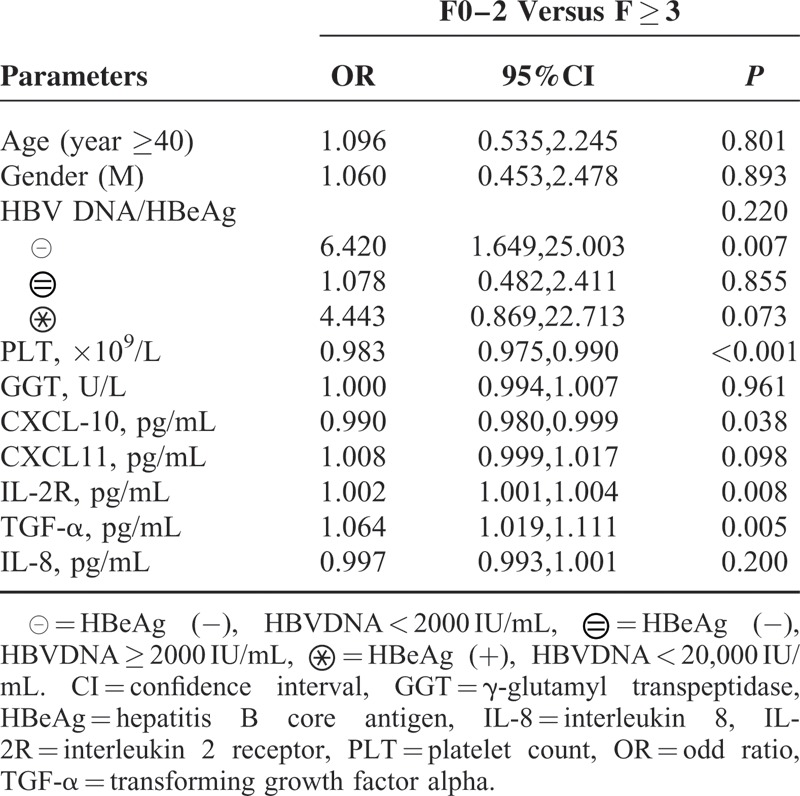
Multivariate Analysis of Clinical Parameters, Cytokines With Liver Fibrosis in Total Patients (n = 227)

### Cytokines as Predictors for Moderate Inflammation in Patients With ALT Less Than 2 × ULN

For patients with ALT < 2 × ULN, clinical parameters such as age, gender, BMI, HBV DNA/HBeAg status, ALP, and TBil were not correlated with liver inflammation. PLT was significantly lower, and AST and GGT were higher in patients with at least moderate inflammation. Within cytokines and chemokines, only serum CXCL-11 was significantly increased with increased HAI scores (*P* < 0.05). Multivariate analysis indicated that CXCL-11, CXCL-10 (*P* = 0.053), AST, GGT, and HBeAg/HBV DNA were independently associated with moderate or severe inflammation. However, *P* values for CXCL-10, GGT, and AST were not significant (*P* > 0.05) (Table 4).

**TABLE 4:**
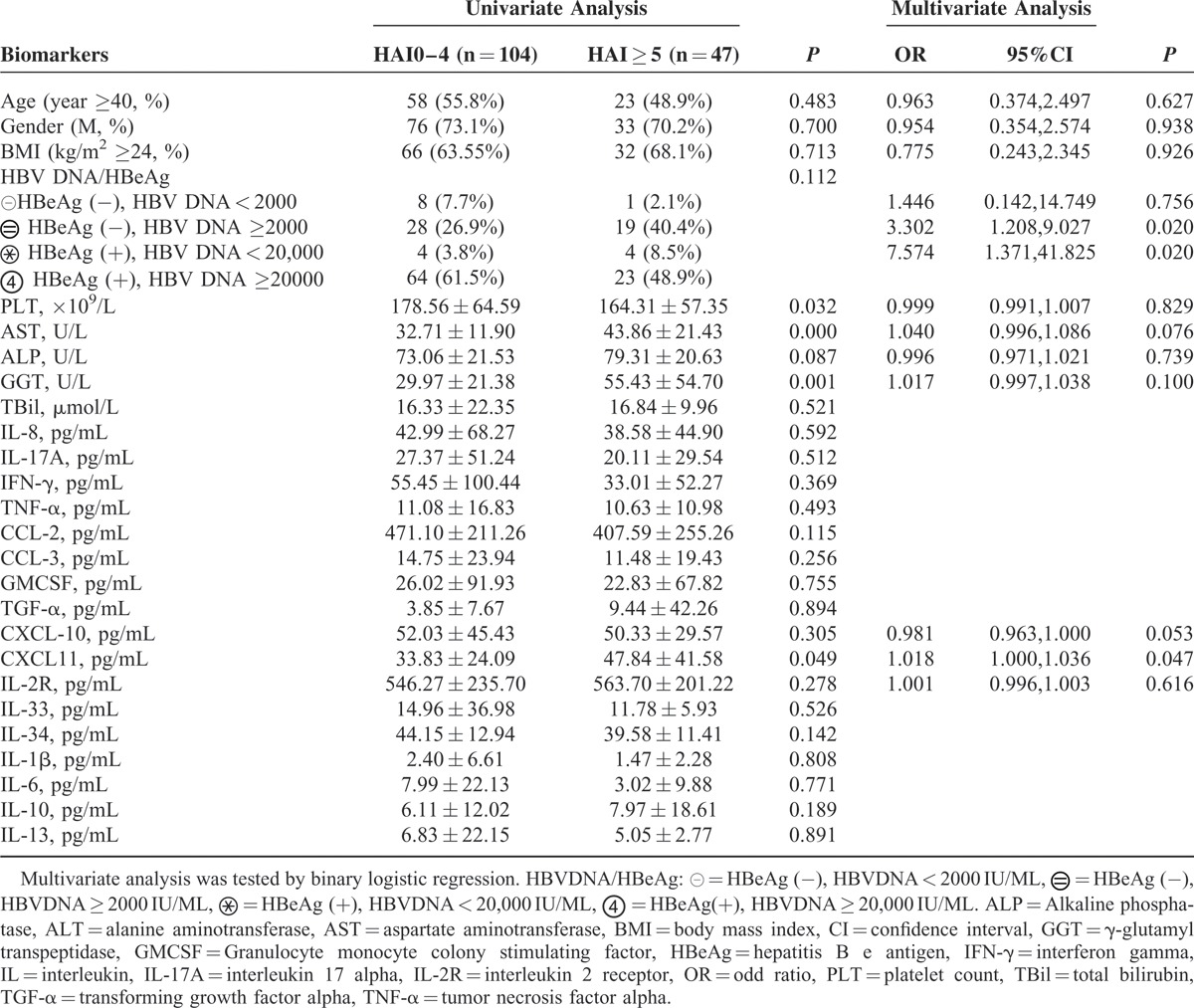
Univariate and Multivariate Analysis of Clinical Parameters, Cytokines With Histological Activity Index in Patients With ALT < 2 × ULN (n = 151)

Based on the variables above, we developed an inflammation-index by binary logistic regression.

 
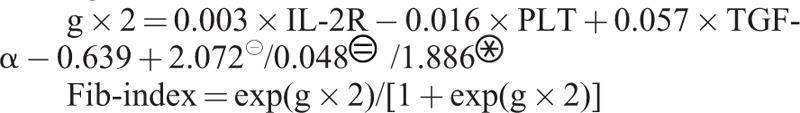


Inflammation-index = exp(g× 1)/[1 + exp(g× 1)]

### Cytokines as Predictors for Significant Fibrosis in Patients With ALT Less Than 2 × ULN

For patients with ALT < 2 × ULN, clinical parameters such as age, gender, BMI, HBV DNA/HBeAg status, ALP, and TBil were not correlated with significant fibrosis stages. PLT was significantly lower, and AST and GGT were higher in patients with significant fibrosis. Serum levels of IL-8 (44.80 ± 49.87 pg/mL, *P* = 0.043), TNF-α (13.48 ± 16.41 pg/mL, *P* = 0.060), CXCL-10 (60.49 ± 41.40 pg/mL, *P* = 0.007), IL-2R (633.55 ± 257.71 pg/mL, *P* = 0.004), and TGF-α (11.90 ± 44.12 pg/mL, *P* = 0.041) were higher in patients with significant fibrosis than in those without. Multivariate analysis showed TGF-α, IL-2R, platelets, and HBeAg/HBV DNA were independent predictors for significant fibrosis in patients with ALT < 2 × ULN (Table 5).

**TABLE 5:**
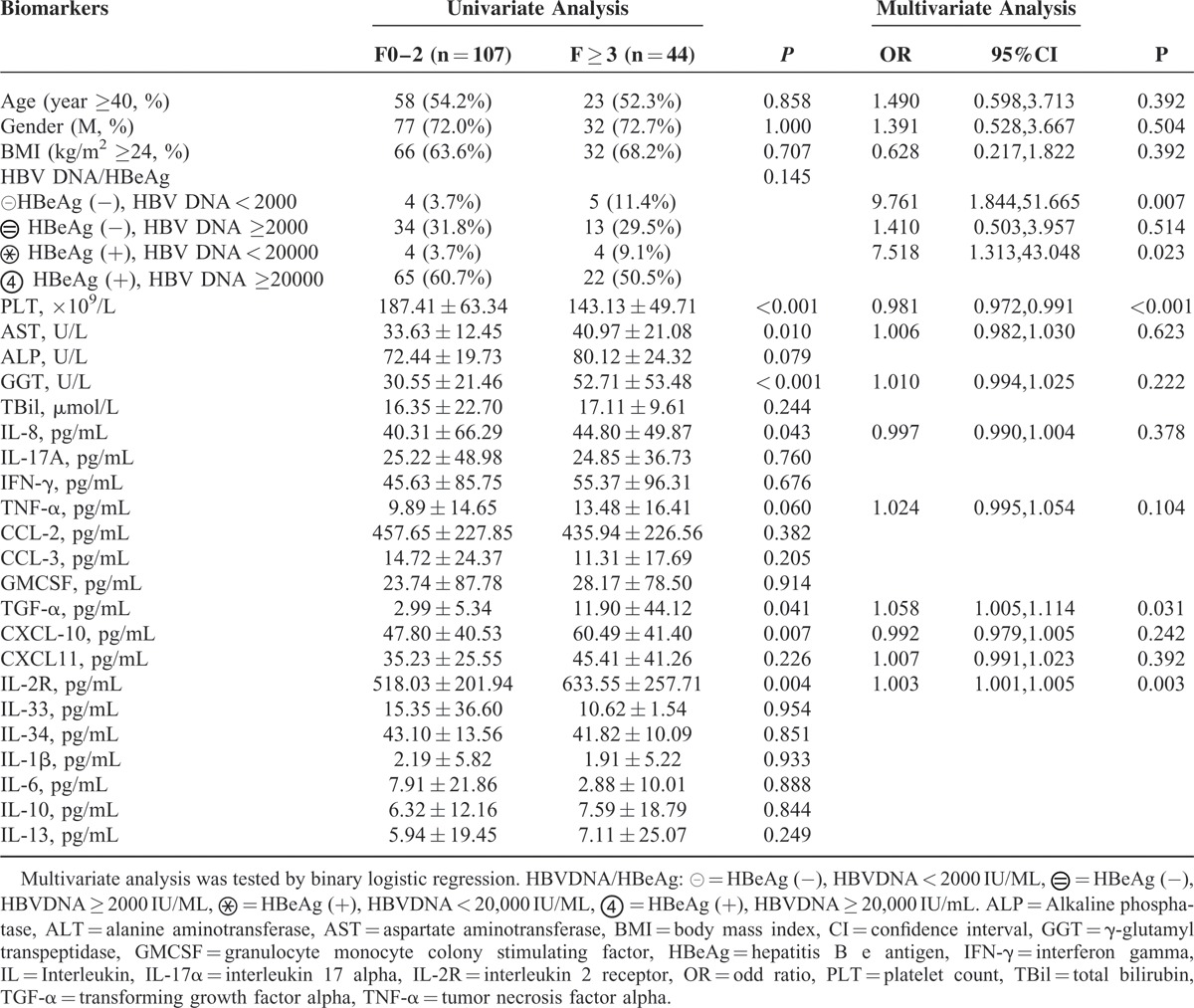
Univariate and Multivariate Analysis of Clinical Parameters, Cytokines With Liver Fibrosis Stages in Patients With ALT < 2ULN (n = 151)

Fib-index was developed to predict significant fibrosis by binary logistic regression, using TGF-α, L-2R, platelet, and HBeAg/HBV DNA as variables.

 
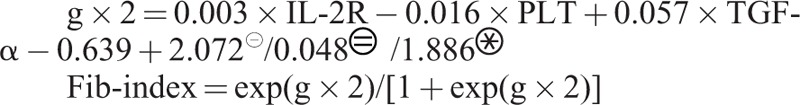


Fib-index = exp(g × 2)/[1 + exp(g × 2)]

### Predictive Performance of Cytokine-Based Scores for Moderate or Severe Inflammation and Fibrosis in Patients With ALT < 2 × ULN

**FIGURE 3:**
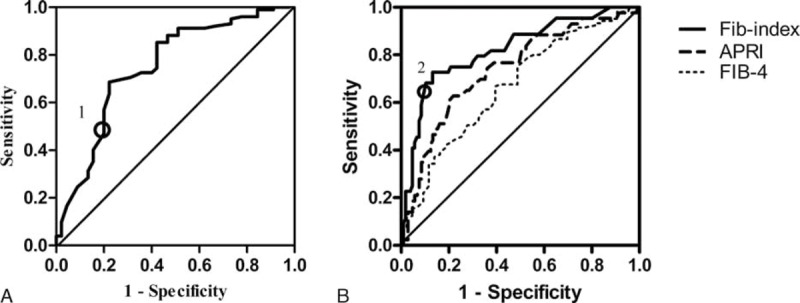
Receiver operating characteristics analysis showing the predictive value of inflammation-index for moderate inflammation (HAI ≥ 5) and fib-index for significant fibrosis (F ≥ 3) in patients with ALT < 2 × ULN. (A) ROC curve for inflammation-index. Circles mark cutoff values of inflammation-index (marked 1: cutoff at 0.46). (B) ROC curve for fib-index, AST to platelet ratio index (APRI), FIB-4 score. Circles mark cutoff values of fib-index (marked 2: cutoff at 0.44). ALT = alanine aminotransferase, AST = aspartate aminotransferase, HAI = histological activity index, ROC = receiver operating characteristic curves, ULN = upper limit of normal range.

The inflammation-index had an areas under the receiver operating characteristics curve (AUROC) of 0.75 (95% CI 0.66–0.84) for diagnosing moderate or greater inflammation in patients with ALT < 2 × ULN. Using a cutoff of ≥0.46, moderate or greater inflammation could be diagnosed with a sensitivity of 48.9%, specificity of 91.2%, NPV of 80.2%, PPV of 71%, and accuracy of 78.2%. Fib-index, APRI, and FIB-4 showed AUCs of 0.82 (95% CI 0.75, 0.90), 0.74 (95% CI 0.65, 0.83), and 0.67 (95% CI 0.57, 0.76), respectively, for diagnosing significant fibrosis in patients with ALT < 2 × ULN. Applying a cutoff of ≥0.44, significant fibrosis could be diagnosed by fib-index with sensitivity of 63.6%, specificity of 90.6%, NPV of 85.7%, PPV of 73.7%, and accuracy of 82.7% (Fig. 3).

To validate these noninvasive models for predicting moderate or greater inflammation and significant fibrosis, LOOCV was performed. LOOCV showed that 74.5% cross-validation grouped cases were correctly classified by inflammation-index and 81.3%, 74.3%, and 64.2% classified by fib-index, APRI, and FIB-4, respectively, in patients with ALT < 2 × ULN (Supplemental Table S2, http://links.lww.com/MD/A510).

In the total population, the inflammation-index had an AUC of 0.76 for moderate inflammation, while fib-index, APRI, and FIB-4 showed AUCs of 0.78, 0.66, and 0.63 for significant fibrosis, respectively (Supplemental Table S2, http://links.lww.com/MD/A510).

## DISCUSSION

This study showed that selected circulating cytokines were associated with liver inflammation and fibrosis. This implies that there are likely various mechanisms of liver inflammation and fibrosis during the progression of chronic HBV infection. Previous studies have shown that cytokines initiate downstream signaling pathways by binding to specific receptors expressed on the target cells, and which subsequently secrete various cytokines.^26^ However, the exact mechanisms by which cytokines mediate liver inflammation and fibrogenesis are not fully understood. INF-γ is well known to be the main mediator for control of HBV infection.^8^ In the progression to HBV-induced inflammation, INF-γ may be overproduced to clear HBV infection, but may be exhausted in the late stages of chronic inflammation. The inability to control the viral infection leads to the recruitment of inflammatory infiltrates into the liver parenchyma by IFN-γ-inducible CXCL-10 and -11 chemokines.^27^ Some of the results in the current study could be explained by the fact that INF-γ showed a declining trend while CXCL-10 and CXCL-11 levels increased in patients with moderate and severe inflammation compared to those without.

The current study also showed that circulating IL-8, TGF-α, and IL-2R levels were increased in patients with significant fibrosis. IL-8 has been proven to play a role in recruitment and activation of hepatic macrophages by CXCR1 in human liver cirrhosis.^28^ TGF-α is a ligand for the epidermal growth factor receptor, which is active in many biological processes, and is a central mediator in the initiation and maintenance of fibrosis in many diseases.^29^ In in-vitro studies, TGF-α has been found to attenuate hepatic fibrosis through upregulation of MMP-1 and inhibition of type I procollagen peptide synthesis.^30,31^ To the best of our knowledge, this is the first study to demonstrate that circulating TGF-α levels were independently associated with liver fibrosis. IL-2R is primarily secreted by activated T-helper lymphocytes and is widely expressed on activated T lymphocytes, regulatory T cells, B cells, and monocytes.^32^ Some studies have examined IL-2R in chronic liver disease and found increased IL-2R in hepatic cirrhosis.^33–36^ However, the performance of IL-2R in diagnosing liver inflammation and fibrosis had not been determined. In the present study, circulating IL-2R levels increased in both moderate inflammation and significant fibrosis in CHB patients.

Serum ALT is the most widely used parameter to screen for and to monitor patients with liver disease. However, patients with HBV infection, a disease in which apoptosis is likely the dominant mechanism of cell death, can have normal or only minimally elevated serum ALT levels.^37^ A recent meta-analysis concluded that 20.7% of 830 CHB patients with ALT levels ≤40 IU/L had significant fibrosis.^38^ The current study showed that 31.7% and 29.1% of 151 patients with ALT levels <2 × ULN had moderate inflammation and significant fibrosis, respectively. It should be noted that patients with significant fibrosis or moderate/severe necro-inflammation should be treated immediately according to current guidelines.^6,7^ Therefore, the use of serum ALT levels underestimates the proportion of patients who should urgently receive antiviral therapy. There are no reliable noninvasive markers as surrogate for ALT to assess histological liver damage in patients with ALT levels <2 × ULN. In this study, we assessed the relationship between circulating cytokines and liver inflammation and fibrosis in patients with ALT levels <2 × ULN. Our results indicated CXCL-11 as a biomarker independent of other biochemical parameters and known risk factors for moderate greater inflammation. We also found IL-2R and TGF-α to be independent indicators for significant fibrosis.

The APRI and FIB-4 scores derived from patients with HCV infection are the 2 most widely studied noninvasive tools for assessing liver fibrosis in CHB patients.^39,40^ Studies on APRI and FIB-4 have been validated in patients with normal and mildly elevated ALT, showing AUROCs of 0.71 and 0.72, respectively.^41^ We employed the 2 scores in our study to evaluate significant fibrosis, obtaining AUROCs of 0.74 and 0.67. Compared to the existing scores, the fib-index performed better in diagnosing significant fibrosis for patients with ALT < 2 × ULN.

The current study has limitations such as a lack of a validation group. A proper internal validation is necessary for the development of a reliable and reproducible prognostic model for external validation.^42^ Although a validation set was not designed in this study, LOOCV was utilized to detect the reliability of inflammation-index and fib-index. The LOOCV approach has the advantage of producing model estimates easier and with less bias in smaller samples.^43^ Another limitation of our study is lack of longitudinal data; this will require future work. Finally, not all the cytokines which could be detected were measured.

In conclusion, the present study supports the concept that cytokines contribute significantly to the development of liver inflammation and fibrosis in CHB. Various cytokines have been found to be associated with liver inflammation and fibrosis, which implies that there are likely various mechanisms by which liver inflammation and fibrosis occur and immune cells become activated. Cytokines CXCL-11, INF-γ, and IL-17A are associated with liver inflammation, whereas CXCL-10, IL-2R, TGF-α, and IL-8 are correlated with liver fibrosis in chronic HBV-infected patients. In chronic HBV-infected patients with ALT < 2 × ULN, CXCL-11 was a marker of moderate or severe inflammation independent of other biochemical parameters and known risk factors. IL-2R and TGF-α were independent predictors for significant fibrosis. An IL-2R and TGF-α-based score fib-index was superior to the existing scores APRI and FIB-4 for predicting significant fibrosis in chronic HBV-infected patients. This represents a promising tool for non-invasive diagnosis of fibrosis in patients with normal and mildly elevated ALT levels.
